# A machine learning model for predicting surgical intervention in renal colic due to ureteral stone(s) < 5 mm

**DOI:** 10.1038/s41598-022-16128-z

**Published:** 2022-07-11

**Authors:** Miki Haifler, Nir Kleinmann, Rennen Haramaty, Dorit E. Zilberman

**Affiliations:** 1 Department of Urology, Chaim Sheba Medical Center, 52621 Tel Hashomer, Ramat Gan, Israel; 2 Affiliated to the Sackler School of Medicine, Tel-Aviv University, Tel Aviv, Israel

**Keywords:** Urology, Ureter, Urological manifestations

## Abstract

A 75–89% expulsion rate is reported for ureteric stones ≤ 5 mm. We explored which parameters predict justified surgical intervention in cases of pain caused by < 5 mm ureteral stones. We retrospectively reviewed all patients with renal colic caused by ureteral stone < 5 mm admitted to our urology department between 2016 and 2021. Data on age, sex, body mass index, the presence of associated hydronephrosis/stranding on images, ureteral side, stone location, medical history, serum blood count, creatinine, C-reactive protein, and vital signs were obtained upon admission. XGboost (XG), a machine learning model has been implemented to predict the need for intervention. A total of 471 patients (median age 49, 83% males) were reviewed. 74% of the stones were located in the distal ureter. 160 (34%) patients who sustained persistent pain underwent surgical intervention. The operated patients had proximal stone location (56% vs. 10%, *p* < 0.001) larger stones (4 mm vs. 3 mm, *p* < 0.001), longer length of stay (3.5 vs. 3 days, *p* < 0.001) and more emergency-room (ER) visits prior to index admission (2 vs. 1, *p* = 0.007) compared to those who had no surgical intervention. The model accuracy was 0.8. Larger stone size and proximal location were the most important features in predicting the need for intervention. Altogether with pulse and ER visits, they contributed 73% of the final prediction for each patient. Although a high expulsion rate is expected for ureteral stones < 5 mm, some may be painful and drawn out in spontaneous passage. Decision-making for surgical intervention can be facilitated by the use of the present prediction model.

## Introduction

Renal colic due to ureteral stone is among the most common reasons for acute admissions to a hospital urology department. This may be explained by the relatively high lifetime prevalence of ureteral stone, recently estimated at 14%^1^. While there is almost no question about the need for surgical intervention in the case of large ureteric stones, the treatment protocol for stones < 5 mm may vary.

The English literature reports a 71–98% stone expulsion rate for distal ureteral stones < 5 mm^2^ and 75–89% for any ureteral stone ≤ 5 mm^3–5^. The highest odds of spontaneous expulsion are estimated for stones in the distal ureter^5,6^, and they generally increase as the stone location in the ureter lowers^3,4^.

The current European Association of Urology and Canadian Association of Urology guidelines suggest that 95% of the 4–5 mm ureteral stones are expected to pass spontaneously within 40 days^7,8^. The authors of a recent study revealed that most of these stones passed within 4 weeks and at an average of 17 days, and therefore recommended surgical intervention only after 4 weeks of non-expulsion of the small stone(s)^3^. Moreover, two recent studies found that early surgical intervention for stones < 5 mm was not recommended^4,9^ since 30% of early intervention cases for any ureteral stone required subsequent hospital-based care^9^.

Patients with renal colic due to ureteric stones < 5 mm admitted for pain fall between the cracks. The obvious purpose of their admission is to control their pain and render them entirely pain-free for discharge and outpatient follow-up. Some of them, however, will experience pain that will eventually require surgical intervention. In the present study, we sought to identify the parameters that may predict the eventual need for surgical intervention in those cases of persistent pain caused by ureteral stone(s) < 5 mm. Such information may help in guiding management decision-making to either spare a patient from undergoing surgery and hospitalization for a stone that is highly likely to pass spontaneously or, alternatively, to spare a patient from enduring weeks of pain from a stone that is not.

## Patients and methods

Following approval of our institutional review board (Chaim Sheba Medical Center, #SMC-20-6942), we retrospectively reviewed all admissions to our urology department between June 1, 2016 and October 31, 2021 that were coded 788.0 (renal colic) as per the International Classification of Disease, 9th edition, Clinical Modification (ICD9-CM). Out of those admissions, we retrieved all cases of renal colic caused by a ureteral stone < 5 mm according to the medical record. The relevant images (i.e., ultrasound [US] and computerized tomography [CT]) contained in each medical record were re-evaluated by a single fellowship-trained senior endourologist (DEZ). The stone’s maximal diameter was evaluated on the coronal CT plane as described elsewhere^10^. Given the retrospective nature of this study, informed consent has been waived by the Chaim Sheba Medical Center ethics committee.

### Exclusion criteria

Excluded were individuals who were < 18 years of age, had fever on admission (i.e.: body temperature > 38 °C/100.4 °F), pregnant women, and patients with bilateral ureteral or multiple ipsilateral ureteral stones, anatomic anomalies (double complete or incomplete collecting system, horseshoe kidney, pelvic kidney, crossed-ectopic kidney), a concomitant ureteral tumor in the ipsilateral side, a single kidney and previous ureteral surgeries (e.g., re-implantation, segmental ureterectomy, or endoscopic treatment for ureteric stricture).

Skeletal contractures were also an exclusion criterion, given their potential to distort the normal ureteral course and adversely affect stone expulsion.

### Features of data extraction

We conducted a meticulous review of the relevant English literature in order to identify all possible factors that could predict spontaneous stone passage and built our database in accordance with variables that had been associated with stone expulsion or were explored in earlier studies on such a possible association. The variables that were extracted and included in our current analysis were : age and sex^9–14^, body mass index (BMI)^13^, the presence of concomitant diabetes mellitus (DM)/hypertension (HTN)/dyslipidemia^11,12^, history of ureteral stones^11,13^, hydronephrosis on imaging studies^9,10,12,15^, perinephric stranding on imaging studies^10,12,15^, ureteral side^14^, stone location (proximal/distal ureter)^3,5,9,12,14^, serum white blood cell (WBC) count^11,13,16^, neutrophil percentage (polymorphonuclears [PMN])^10,16,17^, creatinine level^10–13^, C-reactive protein (CRP) level^13,17,18^, and length of hospital stay^9^. Additional variables included vital signs on admission (pulse, systolic/diastolic blood pressure) as well as the total number of visits to the emergency room before the index admission.

### Clinical management

All of the study patients had been admitted to our urology department due to pain associated with a < 5 mm ureteral stone. The aim of the admission was pain control. Based upon the findings reported by Pickard et al.^19^, none of our patients received medical expulsive therapy of any kind. The patients who had been successfully treated conservatively were scheduled for a follow-up visit in our outpatient clinic with repeat US and serum creatinine level. The aim of that visit was to confirm stone expulsion and schedule another visit when it had not occurred or when the findings were not clear. Patients experiencing intractable pain—i.e.: irresponsive or partially responsive to various pain medications (including IV opiates) and did not enable home discharge—subsequently underwent in-house surgical intervention, preferably primary ureteroscopy and, whenever impossible at that time (due to tight ureter), they underwent a ureteral JJ-stent insertion procedure followed by definitive surgery at a later time.

### Further considerations

Time to surgery or time to expulsion was calculated from the first presentation of renal colic^11^. The date of stone expulsion was defined as either the day on which the patient reported stone expulsion or the day an imaging study ruled out the presence of an obstructing stone. In our country, health system is public and centralized in an electronic health system that captures all visits in other healthcare services including auxiliary images, blood tests and surgical procedures preformed outside our hospital even if the patient failed to show up for a follow-up visit.

### Statistical analysis

Data are presented as frequencies (percentages) for categorical variables or as median (interquartile range [IQR]) for continuous variables. Continuous variables were compared with the Wilcoxon rank-sum test. Categorical variables were compared with the Fisher exact test. Missing data were imputed with multivariate imputation via chained equations^20^. The entire cohort was randomly split into a training set (80%) and a test set (20%). Extreme Gradient Boosting (XG Boost, XG) is a machine learning algorithm for regression and classification which uses an ensemble of weak prediction models, typically decision trees. As an ensemble tree model, XG uses multiple iterative gradient boosters to construct a strong classification system. As a non-linear classifier, XG is able to capture non-linear relations between features, which logistic regression cannot. XG is fast, provides high performance and has been used extensively in the medical field. We used XG with tenfold cross validation to train the model with the training set. An automated grid search was performed to identify the optimal hyperparameters of the model. Model performance was assessed with the Receiver – Operating characteristic (ROC) curve. The optimal probability cut-point for predicting the need for intervention was assessed with the Youden's index. Shapley Additive Explanation (SHAP) scores were used to identify each variable influence on the final prediction^21^. We reported the SHAP score in units of change in prediction probability and defined them as ‘variable importance’. Statistical analyses were performed with R v.3.6.1: R Foundation for Statistical Computing (Vienna, Austria). Multiple comparisons were accounted for by using the false detection rate^22^. All tests were 2-sided, with significance considered at *p* < 0.05.

### Ethics approval

The present study was performed in accordance with the declaration of Helsinki and was approved by Chaim Sheba Medical Center ethics committee, approval # SMC-20-6942. Given the retrospective nature of this study, informed consent has been waived by the above mentioned committee.

## Results

Patients' demographics are described in Table 1. A total of 471 patients were included in this study. Their median age was 49 (41–60) years and 83% were males. Of them, 303 (64%) had no history of a ureteral stone. The majority of the stones (74%) were located in the distal ureter, and stone diameter was 3.5 (3–4.1) mm. A proximal stone location was more common in patients who needed an intervention (56% vs. 10%, respectively, *p* < 0.001). The operated patients had larger stones (4 mm vs. 3 mm, *p* < 0.001), longer length of stay (3.5 days vs. 3 days, *p* < 0.001) and more emergency room visits prior to index admission (2 vs. 1, *p* = 0.007) compared to those who had no surgical intervention. Time to surgical intervention or stone expulsion was not significantly different (7 vs. 10 days, *p* = 0.3).

**Table 1:** Demographics and clinical features.

	Overall, N = 471^a^	Yes, N = 160^a^	No, N = 311^a^	*p* value
Age [Years]^c^	49.0 (41.0, 60.0)	51.5 (40.0, 61.0)	49.0 (41.0, 60.0)	0.4
Sex [n(%)]^b^				0.5
Male	391 (83%)	130 (81%)	261 (84%)	
Female	80 (17%)	30 (19%)	50 (16%)	
BMI [Kg/m^2^]^c^	27.3 (24.6, 30.7)	27.4 (24.7, 31.2)	27.2 (24.5, 30.2)	0.3
DM [n(%)]^b^	70 (15%)	26 (16%)	44 (14%)	0.5
HTN [n(%)]^b^	93 (20%)	37 (23%)	56 (18%)	0.2
Hyperlipidemia [n(%)]^b^	65 (14%)	23 (14%)	42 (14%)	0.8
First ipsilateral stone event [n(%)]^b^	303 (64%)	107 (67%)	196 (63%)	0.4
**Imaging findings**				
Hydronephrosis [n(%)]^b^	406 (86%)	141 (88%)	265 (85%)	0.4
Perinephric Stranding [n(%)]^b^	293 (62%)	100 (62%)	193 (62%)	> 0.9
Stone location [n(%)]^b^				**< 0.001**
Distal Ureter	350 (74%)	71 (44%)	279 (90%)	
Proximal Ureter	121 (26%)	89 (56%)	32 (10%)	
Stone Size [mm]^c^	3.5 (3.0, 4.1)	4.0 (3.3, 4.4)	3.0 (2.4, 4.0)	**< 0.001**
Stone Side [n(%)]^b^				0.6
Left	258 (55%)	85 (53%)	173 (56%)	
Right	213 (45%)	75 (47%)	138 (44%)	
**Laboratory findings**				
WBC [K/microL]^c^	11.3 (9.0, 14.1)	11.1 (8.8, 13.3)	11.3 (9.1, 14.1)	0.3
PMN [%]^c^	76.8 (69.3, 83.0)	76.8 (69.3, 83.0)	76.7 (69.3, 82.5)	0.7
Creatinine [mg/dL]^c^	1.4 (1.1, 1.6)	1.4 (1.1, 1.7)	1.4 (1.1, 1.6)	0.4
CRP [mg/l]^c^	21.2 (6.2, 71.6)	23.4 (6.6, 73.4)	19.8 (6.0, 70.4)	0.5
**Administrative data**				
Pulse [1/min]^c^	78.0 (69.0, 89.0)	77.5 (68.0, 88.0)	78.0 (69.0, 89.0)	0.9
Systolic BP [mmHg]^c^	143.0 (127.0, 157.0)	142.5 (125.8, 155.0)	144.0 (128.0, 158.0)	0.2
Diastolic BP [mmHg]^c^	84.0 (76.0, 93.0)	83.0 (75.8, 90.0)	84.0 (76.0, 94.0)	0.13
Length of hospital stay [Days]^c^	3.0 (2.0, 4.0)	3.5 (3.0, 5.0)	3.0 (2.0, 4.0)	**< 0.001**
ER visits before admission^c^	1.0 (1.0, 2.0)	2.0 (1.0, 2.0)	1.0 (1.0, 2.0)	**0.007**
Time from symptom onset to stone expulsion/removal [Days]^c^	9.0 (5.0, 18.0)	7.0 (5.0, 18.0)	10.0 (5.0, 18.5)	0.3

^a^Median (IQR); n (%).

^b^Categorical variable, Fisher exact test.

^c^Continuous variable, Wilcoxon rank sum test.

Bold indicates significant. Yes = Surgically Treated; No = Conservative Treatment.

None of the other evaluated variables were significantly different across the intervention groups.

**Figure 1:**
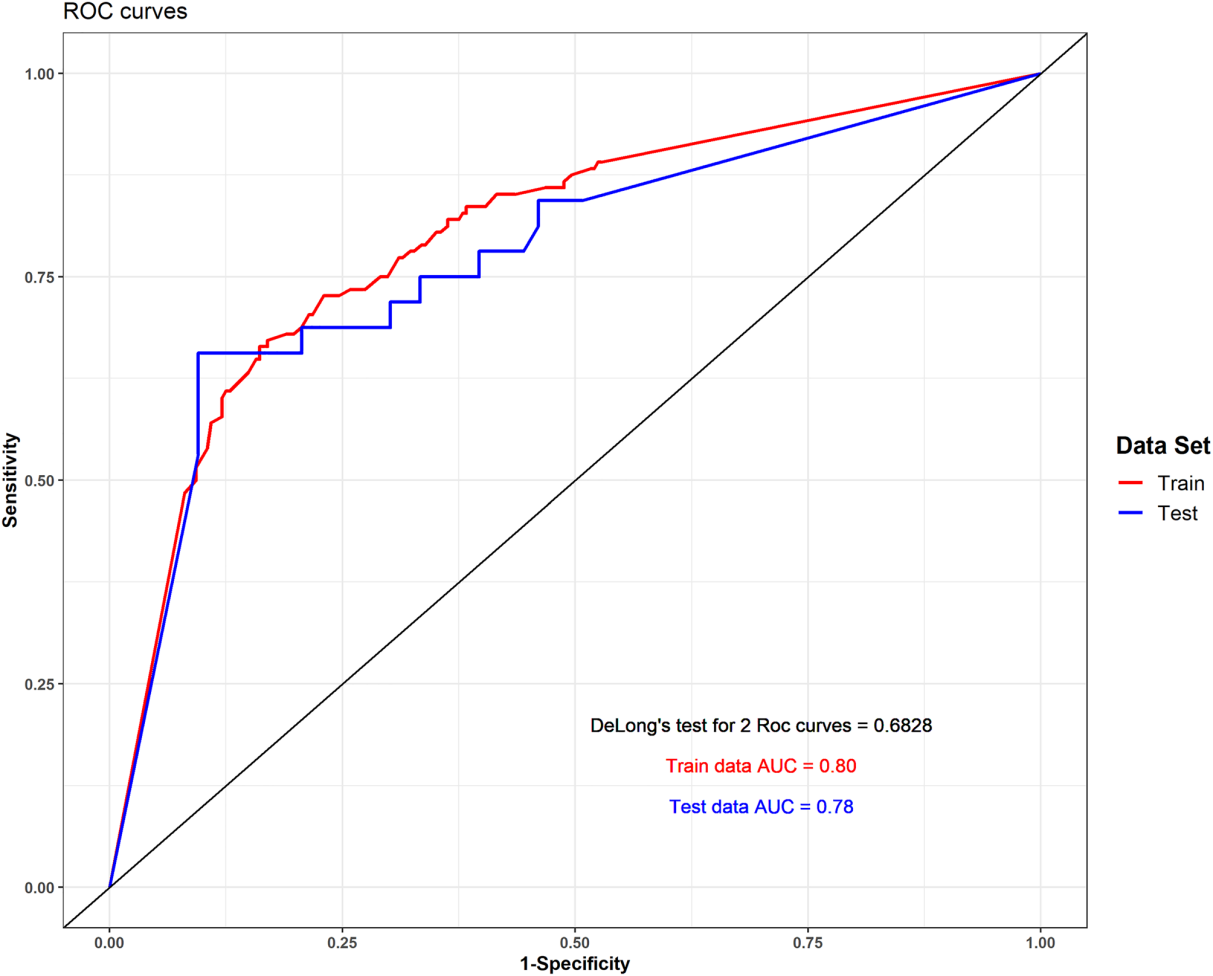
Receiver-operator characteristic curve of the regression model. AUC—area under the curve.

**Figure 2:**
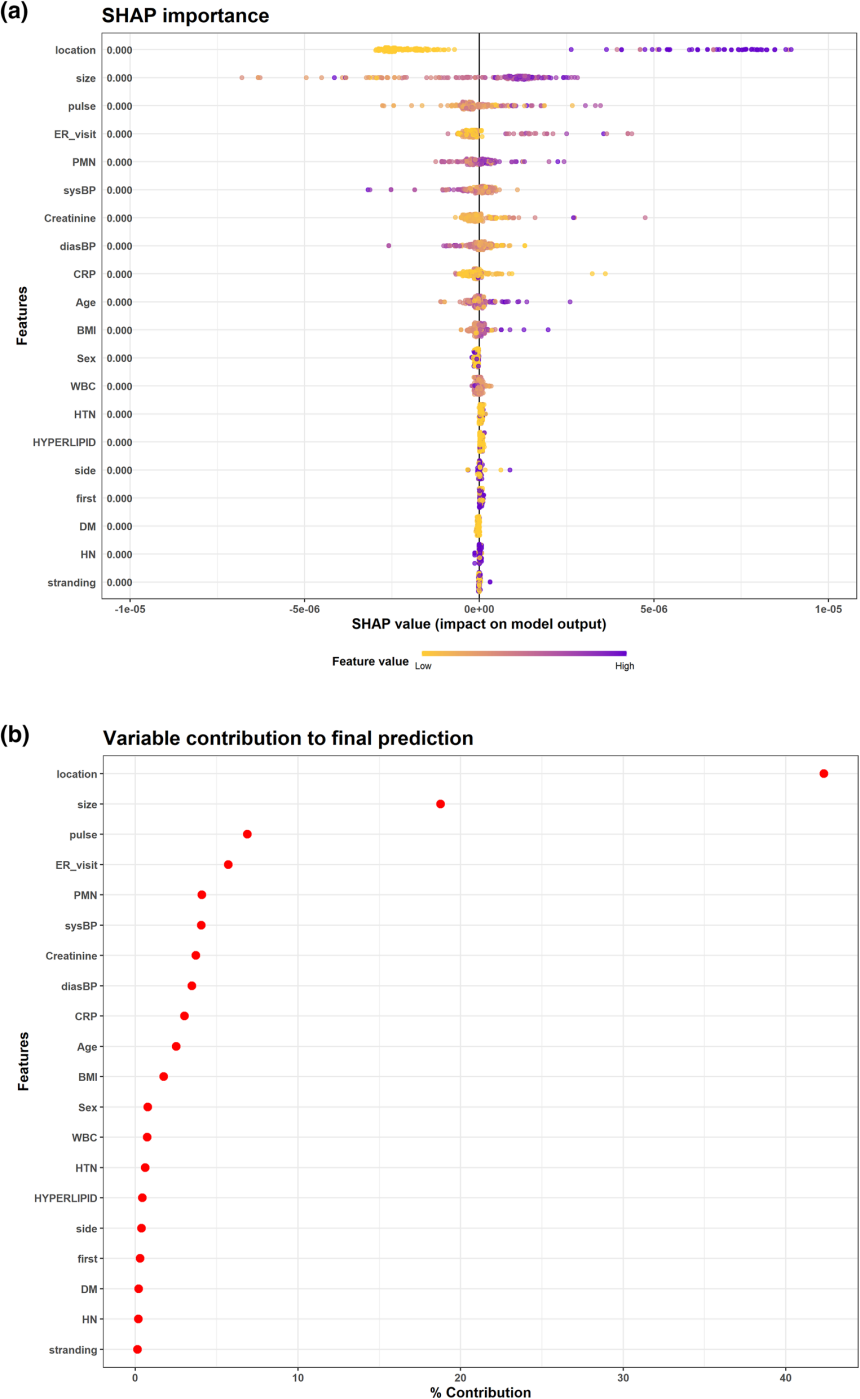
Feature importance of the XG model. (**a**) SHAP values. Each dot represents a patient measurement. The figure depicts the change in prediction probability when changing a feature value. Wider range depicts higher impact on the prediction. (**b**) Mean feature importance. Each dot represents the features contribution to the final prediction of the model. Higher contribution represents more impact on the final prediction.

The XG model was trained to classify patients who would and would not need surgical intervention based on their clinical features. The XG model accurately predicted the need for intervention in the training and test sets (AUC 0.8 and 0.78 respectively, Fig. 1). The optimal probability cut-point in the test set was 0.5. The accuracy, sensitivity and specificity for this cut-point were 0.88, 0.85 and 0.88 respectively. The confusion matrix with number of correct and wrong intervention prediction is depicted in Table 2. The most important features for intervention prediction were stone location and size. Specifically, proximal and larger stones increased the probability of surgical intervention. Furthermore, pulse and number of ER visits were also influential on the probability of the need for intervention. Those 4 features contributed 73% of the final prediction for each patient. Other features had smaller impact on prediction (Fig. 2).

**Table 2:** Confusion matrix for the optimal probability threshold (Youden's index).

Intervention prediction		
	Predicted label	
	Intervention	No intervention
**True label**		
Intervention	56	8
No intervention	14	111

**Figure 3:**
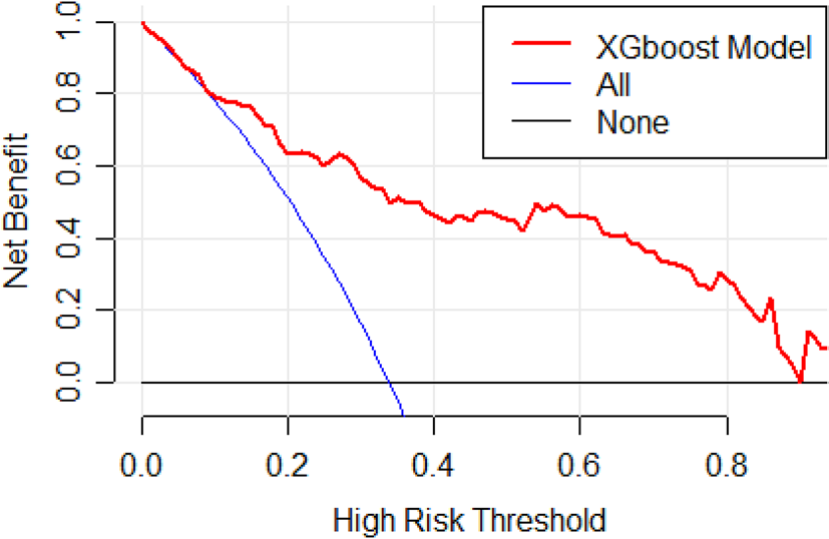
Decision curve depicting the net benefit obtained by using the regression model for different probability thresholds for recommending an intervention. The model demonstrates higher clinical net benefit compared to the default strategies across reasonable threshold probabilities.

The decision curve and clinical benefit of the model are depicted in Fig. 3. Our model demonstrated a significant clinical benefit across all probability thresholds.

## Discussion

Ureteral stones < 5 mm are highly likely to undergo spontaneous expulsion, and the lower they are along the ureter, the higher their chance to pass spontaneously^3^. This observation has dictated the conservative management recommended by urological professional associations for ureteral stones < 5 mm^7,8^. Nevertheless, small ureteral stones may cause persistent pain, and despite the promising expulsion statistics, it may be challenging to decide not to operate in cases that do not respond whatsoever to analgesics. Given the potential adverse effects of early surgical intervention^4,9^, these cases should be carefully selected. It is, therefore, of considerable importance to know what information in their initial clinical data can be used to identify the patients with pain due to any ureteral stone < 5 mm who will most likely require surgical intervention and undertake it without unnecessary delay.

### Demographics

Age has been considered to negatively affect stone expulsion^9,13^ presumably due to decreased ureteral peristalsis with aging^13^. That correlation, however, was not confirmed by the results of the present study nor by those of others^4,10–12,14^. recent study found that females tend to undergo a surgical intervention more often than males^9^, however many studies, including the present one, failed to demonstrate that finding^4,10–14^.

BMI was rejected as a predicting factor for surgical intervention by us as well as by others^13^.

HTN, DM and dyslipidemia had no influence on the surgical intervention rate. Similar to us, Choi et al.^12^ failed to demonstrate any correlation between HTN and stone expulsion rate, however, their patients with DM were shown to have a better chance to pass their stones. The small number of their patient population (n = 26) calls for caution in accepting that conclusion.

Three studies examined previous spontaneous stone passage (SSP) and the likelihood of surgical intervention, and reached 3 different conclusions. While one study, similar to our findings, found no correlation^10^, another observed that a past history of nephrolithiasis directly correlated with higher odds for surgical intervention^11^. A third study reported a negative association between a previous SSP and the likelihood of surgical intervention^13^.

### Imaging findings

Hydronephrosis has been widely investigated as a possible predictor of surgical intervention for ureteral stones < 10 mm. Hydronephrosis was more frequent in patients who had undergone surgery in some studies^4,9,13^ but neither in our study nor in others^10,12^.

As was the case in previous studies^4,10,12^, we ruled out perinephric stranding as a predicting factor for surgical intervention.

The right ureter had been associated with a higher likelihood of spontaneous expulsion^14^, but neither we nor others^10,12^ found that to be the case.

There is a consensus among all studies that explored the odds that stone size^4,5,9,10,12–14^ and location^3,4,9–12,14^ strongly correlated to the likelihood of spontaneous ureteral stone expulsion. In the case of distal ureteral stones 4–10 mm in size the larger the stone, the lesser the chances that it will pass spontaneously^13^. Another study set the threshold at a stone size of 4 mm in diameter, namely, the larger a distal ureteral stone above this threshold, the less likely it will pass spontaneously^10^. Interestingly, as demonstrated in our study, this applied even to cases of stones < 5 mm, which are allegedly high likely to expulse.

In the present study, patients who had undergone surgical intervention were more likely to have their stones located in the upper ureter. This finding is in line with previous studies^9,11^ that found the proximal ureteral location as being a strong predictor for conservative treatment failure. Similarly, a lower SSP was reported for stones located in the proximal ureter (25.4–52%) compared to the mid- and distal ureter^3,4,12^. Moreover, in general, the lower the stone, the higher the likelihood of SSP^4,5,10,14^.

### Laboratory findings

The correlation between high levels of serum WBC, PMN and CRP and the likelihood of SSP has been extensively investigated with equivocal findings. Two studies found that high levels of both WBC and CRP were directly correlated with greater chance of surgical intervention^13,18^. This was attributed to inflammatory response around the obstructing stone^13,15^. Moreover, a high serum PMN level was found to be associated with a greater likelihood of surgical intervention^17,18^. On the other hand, 2 other studies reported the opposite, observing that higher levels of both WBC and PMN predicted a higher likelihood of SSP^4,16^. We, like others, found no correlation between serum WBC^10^, PMN^10^ or CRP^4^ levels and the likelihood of surgical intervention. In addition, the serum creatinine level was not observed by us or by others^4,10,11^ as being a predicting factor for surgical intervention.

### Administrative information

Symptoms lasting over 3 days^11^ or 4 days^10^ correlated with a higher likelihood of surgery. Our patients who had undergone surgery, similar to those of another study^9^, were more likely to have a longer admission, although that factor could not serve in the present model as a predicting factor.

Alternatively, the number of ER visits prior to index admission as well as pulse rate on admission served as additional predictors for surgical intervention. We believe that they reflect pain intensity and the consequent need for surgical intervention.

The natural history of ureteral stones < 10 mm has been extensively investigated. To the best of our knowledge, this is the first study that focuses upon ureteral stones < 5 mm that are anywhere in the ureter, and that draws any conclusions with regard to the need for surgical intervention in cases essentially deemed appropriate for conservative management. The relatively large population of patients in the present study further supports its conclusions. Our prediction model has moderate-high accuracy in predicting the need for intervention (AUC-0.8), which is similar to other clinical prediction tools used in urology (i.e.: PSA and MRI for significant prostate cancer detection, 0.78 and 0.82, respectively^23,24^).

Since the threshold probability for intervention depends upon many factors that involve the patient, clinician and availability of hospitalization and surgical facilities, this model provides substantial clinical benefit compared to default strategies (e.g., treat all, treat none) over a wide range of likely threshold probabilities.

We are aware that these data derive from a tertiary care, high-volume center composed of highly experienced fellowship-trained teams and high availability of operating theatres. Under such conditions, the benefit of surgical intervention in selected cases outweighs its risks, a fact that is reflected in a relatively high proportion of surgical interventions in our cohort (34%). This may not be the case in other medical care facilities where team and facility availabilities are limited.

External validation may be required before this model can be implemented in clinical use.

In summary, although a high expulsion rate is expected for ureteral stones < 5 mm, some may be markedly painful and late to pass spontaneously. We determined that accounting for the 4 parameters of stone size and proximal stone location altogether with pulse rate and number of ER visits prior to index admission may facilitate decision making before embarking upon or postponing surgical intervention.

## Data Availability

The datasets used and/or analysed during the current study are available from the corresponding author on reasonable request.

## Abbreviations

US: Ultrasound
CT: Computerized tomography
BMI: Body mass index
DM: Diabetes mellitus
HTN: Hypertension
WBC: White blood cell
PMN: Polymorphonuclear cells
CRP: C-reactive protein
IQR: Interquartile range
XG Boost: Extreme gradient boosting
AUC: Area under the curve
SSP: Spontaneous stone passage
